# Brain-wide slowing of spontaneous alpha rhythms in mild cognitive impairment

**DOI:** 10.3389/fnagi.2013.00100

**Published:** 2013-12-27

**Authors:** Pilar Garcés, Raul Vicente, Michael Wibral, Jose Ángel Pineda-Pardo, Maria Eugenia López, Sara Aurtenetxe, Alberto Marcos, Maria Emiliana de Andrés, Miguel Yus, Miguel Sancho, Fernando Maestú, Alberto Fernández

**Affiliations:** ^1^Laboratory of Cognitive and Computational Neuroscience (UCM-UPM), Centre for Biomedical TechnologyMadrid, Spain; ^2^Department of Applied Physics III, Faculty of Physics, Complutense University of MadridMadrid, Spain; ^3^MEG Unit, Brain Imaging Center, Goethe UniversityFrankfurt, Germany; ^4^Max-Planck Institute for Brain ResearchFrankfurt, Germany; ^5^Institute of Computer Science, Faculty of Mathematics and Computer Science, University of TartuTartu, Estonia; ^6^Department of Basic Psychology II, Complutense University of MadridMadrid, Spain; ^7^Neurology Department, San Carlos Clinical HospitalMadrid, Spain; ^8^Memory Decline Prevention Center, Ayuntamiento de MadridMadrid, Spain; ^9^Radiology Department, San Carlos Clinical HospitalMadrid, Spain; ^10^Department of Psychiatry and Medical Psychology, Faculty of Medicine, Complutense University of MadridMadrid, Spain

**Keywords:** mild cognitive impairment, magnetoencephalography, alpha peak, slowing, hippocampal volume

## Abstract

The neurophysiological changes associated with Alzheimer's Disease (AD) and Mild Cognitive Impairment (MCI) include an increase in low frequency activity, as measured with electroencephalography or magnetoencephalography (MEG). A relevant property of spectral measures is the alpha peak, which corresponds to the dominant alpha rhythm. Here we studied the spatial distribution of MEG resting state alpha peak frequency and amplitude values in a sample of 27 MCI patients and 24 age-matched healthy controls. Power spectra were reconstructed in source space with linearly constrained minimum variance beamformer. Then, 88 Regions of Interest (ROIs) were defined and an alpha peak per ROI and subject was identified. Statistical analyses were performed at every ROI, accounting for age, sex and educational level. Peak frequency was significantly decreased (*p* < 0.05) in MCIs in many posterior ROIs. The average peak frequency over all ROIs was 9.68 ± 0.71 Hz for controls and 9.05 ± 0.90 Hz for MCIs and the average normalized amplitude was (2.57 ± 0.59)·10^−2^ for controls and (2.70 ± 0.49)·10^−2^ for MCIs. Age and gender were also found to play a role in the alpha peak, since its frequency was higher in females than in males in posterior ROIs and correlated negatively with age in frontal ROIs. Furthermore, we examined the dependence of peak parameters with hippocampal volume, which is a commonly used marker of early structural AD-related damage. Peak frequency was positively correlated with hippocampal volume in many posterior ROIs. Overall, these findings indicate a pathological alpha slowing in MCI.

## Introduction

Mild Cognitive Impairment (MCI) is often considered a prodromal stage of Alzheimer's Disease (AD). This is due to the fact that some studies have found that around 10–15% of MCI patients annually progress to AD while it only occurs at a 1–4% rate for the healthy aged population (Petersen, 2001; Petersen and Negash, 2008). MCI patients show objective cognitive alterations but not severe enough to meet the criteria for dementia. In the past years, a great interest has been drawn to MCI, since this condition might help to understand the neurological basis of the “predementia” stages of AD and to maximize the effect of the current available treatments.

Particularly, electrophysiological rhythms have been found relevant in pathological aging. Electroencephalographic (EEG) and magnetoencephalographic (MEG) studies have shown a slowing of the oscillatory rhythms in AD (Berendse et al., 2000; Huang et al., 2000). MCI patients exhibit a reduced mean frequency score in MEG power spectra (Fernández et al., 2006), indicating that the AD-related oscillatory slowing may have its onset in the predementia stage. Additionally, specific spectral profiles have been considered as pathological biomarkers. For example, an increased delta and a decreased alpha1 power were found to be related to a lower cortical gray matter volume (Babiloni et al., 2013). It has also been reported that changes in the high alpha/low alpha ratio or in the theta/gamma ratio are associated with the cognitive status, conversion to AD, hippocampal and amygdalar atrophy or gray matter changes (Moretti et al., 2009a, 2011, 2012).

An essential property of the electrophysiological spectra is the dominant alpha rhythm or alpha peak. Alpha oscillations have been measured over wide regions of the exposed human cortex (Jasper and Penfield, 1949). Sensor-level EEG studies have found that their frequency rises from childhood to adolescence or young adulthood, and then decreases slowly with age (Chiang et al., 2011). Abnormally low alpha peak frequencies can be found in demented patients (Samson-Dollfus et al., 1997). Some studies of MCI have used the posterior dominant frequency to perform spectral analysis. For instance, (Moretti et al., 2009a, 2011, 2012) used the individual alpha peak to define individual frequency ranges for theta, alpha, and beta bands. Babiloni et al. (2009, 2013) considered the alpha peak frequency as a covariate when performing statistical analysis. Nevertheless, although utilized as an intermediate step in the analysis pipeline of many studies, the importance of alpha peak amplitude and frequency values *per se* to define neurophysiological characteristics in MCI has been scarcely investigated.

In the present study we investigated the spatial distribution of resting state alpha peak frequency and amplitude over the whole brain for MCI patients and age-matched healthy controls. To this aim, beamforming was used to estimate MEG spectral parameters for the alpha peak (frequency and amplitude) in source space. Also, we analyzed how these parameters were modulated by age and sex for each ROI. Finally, we examined the relation between peak parameters and hippocampal volume, which is commonly used as a structural biomarker of AD (Dubois et al., 2007).

## Materials and methods

### Subjects

27 patients with a diagnosis of amnestic-MCI and 24 controls were included in this study. Table 1 summarizes their characteristics. MCI patients were recruited at the Geriatric and Neurological Units of the “Hospital Universitario San Carlos,” Madrid, Spain, where they were diagnosed by clinical experts. As introduced in Grundman et al. (2004), inclusion criteria for MCI comprised: (1) memory complaint confirmed by an informant, (2) normal cognitive function, (3) no or minimal impairment in activities of daily living, (4) abnormal memory function, (5) not being sufficiently impaired to meet the criteria for dementia.

**Table 1:** **Subjects characteristics**.

	**Subjects**	**Age (years)**	**Gender (M/F)**	**Educational level**	**MMSE**	**Normalized hippocampal volume**	
						**Left**	**Right**
Control	24	71.8 ± 3.6	6/18	3.8 ± 1.3	29.3 ± 0.9	(2.62 ± 0.37)·10^−3^	(2.59 ± 0.28)·10^−3^
MCI	27	73.9 ± 6.3	14/13	2.7 ± 1.3	27.5 ± 2.2	(2.17 ± 0.41)·10^−3^	(2.08 ± 0.49)·10^−3^

Data are given as mean ± standard deviation.

M = males, F = females. Educational level was grouped into five levels: 1: Illiterate, 2: Primary studies, 3: Elemental studies, 4: High school studies, 5: University studies.

MMSE = Mini Mental State Examination score. Hippocampal volume was normalized with the overall intracranial volume.

Additionally, all subjects were in good health and had no history of psychiatric or neurological disorders. They underwent an MRI brain scan to rule out infection, infarction or focal lesions. Subjects meeting any of the following criteria were excluded from the study: Hachinski score (Rosen et al., 1980) higher than 4, Geriatric Depression Scale score (Yesavage et al., 1982) higher than 14, alcoholism, chronic use of anxiolytics, neuroleptics, narcotics, anticonvulsants, or sedative hypnotics. Additionally, MCI patients underwent an exam to rule out possible causes of cognitive decline such as B12 vitamin deficit, thyroid problems, syphilis, or HIV. Drugs that could affect MEG measurements such as cholinesterase inhibitors were removed 48 h before the MEG scan. The investigation was approved by the local Ethics Committee.

### MEG recordings

Three-minute MEG resting state recordings were acquired at the Center for Biomedical Technology (Madrid, Spain) with an Elekta Vectorview system containing 306 sensors (102 magnetometers and 204 planar gradiometers), inside a magnetically shielded room (Vacuumschmelze GmbH, Hanau, Germany). During the measurements, subjects sat with their eyes closed and were instructed to remain calm and move as little as possible. Each subject's head was digitized in 3D with a Fastrak Polhemus system and four coils were attached to the forehead and mastoids, so that the head position with respect to the MEG helmet was continuously determined. Activity in electrooculogram channels was also recorded to keep track of ocular artifacts.

Signals were sampled at 1000 Hz with an online filter of bandwidth 0.1–300 Hz. Maxfilter software (version 2.2., Elekta Neuromag) was used to remove external noise with the temporal extension of the signal space separation method with movement compensation (Taulu and Simola, 2006).

### MRI acquisition

3D T1 weighted anatomical brain MRI scans were collected with a General Electric 1.5 T magnetic resonance scanner, using a high-resolution antenna and a homogenization PURE filter [Fast Spoiled Gradient Echo (FSPGR) sequence with parameters: TR/TE/TI = 11.2/4.2/450 ms; flip angle 12°; 1 mm slice thickness, a 256 × 256 matrix and FOV 25 cm]. For volumetric analysis, Freesurfer software package (version 5.1.0) and its automated sub-cortical segmentation tool (Fischl et al., 2002) were employed. For the source analysis, the reference system of the T1 volumes was transformed manually using 3 fiducial points and headshape, until a good match between MEG and T1 coordinates was reached.

### Source analysis

Data analysis was done using both FieldTrip software (Oostenveld et al., 2011) and in-house scripts.

#### MEG preprocessing

For the definition of artifact-free epochs, the continuous MEG resting state recording was scanned in non-overlapping segments of 4 s. Segments with ocular, jump, or muscular artifacts were identified and discarded. Per subject, a minimum of 20 artifact-free segments (80 s) remained [controls: (25.7 ± 4.8), MCI: (24.6 ± 6.6)]. After filtering of the continuous original data using a finite impulse response filter of order 1000 and a bandwidth of 1–30 Hz, the artifact-free segments of the data identified in the previous step were extracted for further analysis.

#### Headmodels

First, a regular grid of 2459 points with 1 cm spacing was created in the template Montreal Neurological Institute (MNI) brain. This set of points was transformed to subject's space using a linear normalization between the native T1 image and a standard T1 in MNI space with 2 mm resolution. This grid constituted the source locations. The forward model was solved with a realistic single-shell model (Nolte, 2003).

#### Beamforming

Source reconstruction was performed with Linearly Constrained Minimum Variance beamformer (Van Veen et al., 1997). For each subject, the covariance matrix was averaged over all trials to compute the spatial filter's coefficients, and then these coefficients were applied to individual trials, obtaining a time series per segment and source location. This reconstruction was performed for magnetometers and gradiometers separately, yielding two different source estimates per subject.

### Spectral analysis

Power spectra were obtained from the time series via a multitaper method with discrete prolate spheroidal sequences as tapers and 1 Hz smoothing for frequencies between 2 and 30 Hz, with a 0.25 Hz step. These spectra were averaged over trials and normalized with the sum of the spectral power in the range (2–30) Hz. Then, an average power spectrum per Region of Interest (ROI) and subject was obtained. Eighty-eight ROIs were used in this study and they were defined in MNI space using the Harvard-Oxford probabilistic atlas (Desikan et al., 2006), as implemented in the fMRIb Software Library (FSL) (Jenkinson et al., 2012). Thirty-seven cortical and 7 subcortical ROIs per hemisphere were included (merging subdivisions within gyri in the Harvard-Oxford atlas).

Then, to extract alpha peak parameters, experimental spectra were fitted with a non-linear least-square procedure to:

$$\log(P(f))=B-C\cdot \log(f)+A\cdot \exp\left(\frac{-{\left(f-f_{p}\right)}^{2}}{\Delta^{2}}\right)$$

where A,B,C, Δ, and *f_p_* are adjustable parameters and a wide range (4–13) Hz is used for the fitting. Such a Gaussian peak fit with power-law background has been proven useful for alpha rhythm detection in EEG (Chiang et al., 2008; Lodder and van Putten, 2011).

With this procedure, a peak per ROI was identified separately for the reconstructions based on magnetometers and gradiometers. Then, magnetometer and gradiometer data were combined. Thus, the final peak amplitude and frequency per ROI and subject was calculated by averaging the peak values obtained for both types of sensors. In order to optimize the reliability of the alpha peak estimation, two criteria were considered: Peaks with (1) high inter-trial amplitude variability for any sensor type or (2) a frequency difference between the magnetometer and the gradiometer fit bigger than 1 Hz, were considered spurious and removed from the subsequent statistical analysis.

### Statistical analysis

Peak amplitudes and frequencies were compared with univariate ANOVA tests, separately for each ROI. Shapiro-Wilk and Levene tests were used to ensure normality of the data and equal variances across groups. For the peak amplitude, the transformation *x* → log(*x*/(1 − *x*)) was applied prior to statistical analysis to obtain values following a normal distribution. A four-way ANOVA analysis was performed considering diagnosis, age, sex, and educational level as factors to investigate differences between controls and MCIs and the influence of age and sex on the alpha peak. Finally, we examined whether peak parameters depend on hippocampal volume (which was normalized with the overall intracranial volume). For that, we computed the Pearson correlation coefficient between peak amplitude or frequency and hippocampal volume across all subjects, for every ROI separately. To establish the statistical significance of these correlations, a four-way ANOVA test with hippocampal volume, age, sex, and educational level as factors was used, taking all subjects (Control and MCI) as a single group.

The *p*-values of all ANOVA tests were corrected for multiple comparisons with a procedure based on clustering and permutations, as introduced by Maris and Oostenveld (2007). For that, spatially adjacent ROIs with *p* < 0.05 were first grouped into clusters. Then, the obtained peak values (frequency or amplitude) were 2000 times randomly assigned to the original groups. The sum of *F*-values over each cluster in the original dataset was compared with the same measure in the randomized data. For each cluster, the proportion of randomizations with *F*-values higher than the ones in the original data corresponds to the final *p*-value.

## Results

### Peak fitting

Peaks were successfully identified for most ROIs and subjects, especially in posterior and temporal ROIs. Overall, the peak was harder to find in anterior areas of the brain, since for around 10–15% of the subjects the criteria for robustness introduced before were not fulfilled in frontal ROIs. On the whole, a peak was fit in 80 ± 14 ROIs (given as mean ± std) for the control group and in 85 ± 5 ROIs for the MCI group. For the following ROIs, less than 85% of the subjects showed a robust peak: right paracingulate gyrus, right frontal operculum cortex, right inferior and middle frontal gyri, both superior frontal gyri, both supplementary motor cortices and right pallidum. These ROIs were not considered for statistical analysis. The average peak frequency over all ROIs was 9.68 ± 0.71 Hz for controls and 9.05 ± 0.90 Hz for MCIs and the average normalized amplitude was (2.57 ± 0.59)·10^−2^ for controls and (2.70 ± 0.49)·10^−2^ for MCIs.

### Control vs. MCI

**Figure 1:**
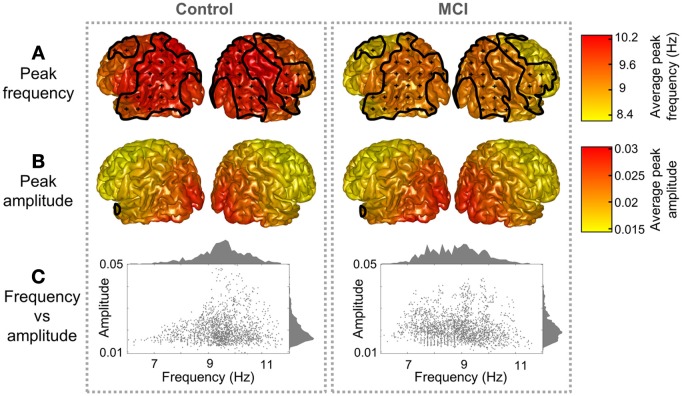
**Peak distribution in controls and MCIs.** Peak **(A)** frequency and **(B)** amplitude grand averages for controls and MCIs. Clusters with significant differences between controls and MCIs (*p* < 0.05) are enclosed with black lines and scattered with black crosses. **(C)** Represents a scatter plot of the peak parameters (frequency and amplitude) for every region and subject. Frequency and amplitude histograms are projected into the y and x axis respectively.

Both groups presented a similar spatial distribution of peak parameters, with higher amplitude and frequency in posterior ROIs, as shown in Figure 1. However, peak frequencies were higher in controls than in MCIs, especially over parietal and temporal ROIs, where differences were statistically significant (*p* < 0.05). Amplitudes were similar in controls and MCIs, although values tended to be higher in MCIs, but this was significant only for six temporal and medial ROIs. As amplitude and frequency values are usually inversely related in electrophysiological power spectra, the amplitude increase in MCIs could be just a consequence of the frequency decrease. To investigate this effect, amplitude values were plotted as a function of frequency (Figure 1C). For controls, amplitudes were higher within the 9–11 Hz frequency range, while for MCIs this range seemed to be broader, with high magnitude alpha peaks from 7 to 11 Hz. On the whole, this leads to the idea that alpha peak frequency is reduced in MCI.

### Age and sex influence

**Figure 2:**
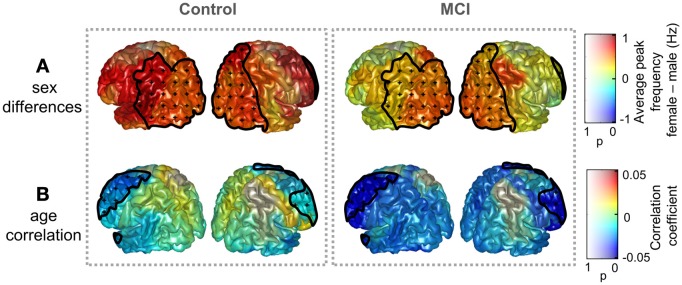
**Influence of age and sex on peak frequency in controls and MCIs. (A)** Peak frequency difference of grand averages: females—males. **(B)** Correlation coefficient between age and peak frequency. Clusters with significant effect of age or sex upon peak frequencies (*p* < 0.05) are enclosed with black lines and scattered with black crosses. Additionally, the *p*-value specifies the transparency of the plotted intensities: a region with *p*-value of 0 shows a full opaque color, whereas a region with *p*-value of 1 will be transparent.

Sex and age did not exert a significant influence on peak amplitude, while significant effects were found for the peak frequency. Figure 2 displays sex differences and age correlations for peak frequency in Controls and MCIs separately. Peak frequency was higher for females than for males both in controls and MCIs. This trend was present over the whole brain, although only statistically significant (*p* < 0.05) over some posterior and right frontal ROIs. Additionally, peak frequency was found to correlate negatively with age. This correlation was strongest in frontal ROIs, where a significant effect (*p* < 0.05) was found.

### Hippocampal volume

**Figure 3:**
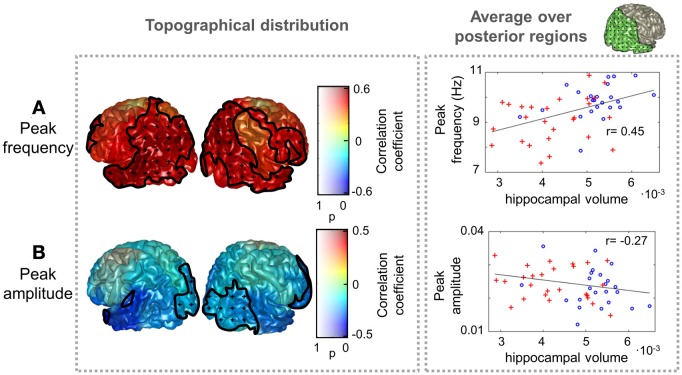
**Peak frequency and amplitude correlations with hippocampal volume.** The distribution of correlation coefficient between peak **(A)** frequency and **(B)** amplitude with hippocampal volume (normalized with intracranial volume) for all subjects (Controls and MCIs) is shown. Clusters with significant effect of hippocampal volume (*p* < 0.05) are marked as in Figure 2. As an example, scatter plots of the average peak frequency and amplitude over posterior ROIs as a function of hippocampal volume are displayed in the right side. The included ROIs are plotted in green in the upper right side of the figure. Controls are represented as blue circles and MCIs as red crosses.

To further assess whether differences in peak parameters could be considered as a pathological sign, the dependence of peak amplitude and frequency values with hippocampal volume was examined. Results are illustrated in Figure 3. Peak frequency correlated positively with hippocampal volume, reaching correlation values up to 0.6, which denote a strong association between both measures. This trend was significant (*p* < 0.05) over most of the postrolandic ROIs of the brain and implies that a slowing in the main alpha rhythm is related with a greater atrophy in the medial temporal lobe. The opposite effect was found for the peak amplitude, which correlated negatively with hippocampal volume over the whole brain, especially over occipital and frontal ROIs, where the trend was significant (*p* < 0.05).

## Discussion

In this paper the alpha peak parameters (frequency and amplitude) were investigated in a sample of MCI patients and controls. Differences between both groups were examined, as well as the influence of age and sex, and the correlation between peak parameters and hippocampal volume. To attain such goal, a novel method was introduced, that combined beamforming for reconstruction of the power spectra in the source space, and a fitting algorithm that has been successfully used for peak identification with scalp EEG measures in sensor space (Chiang et al., 2011; Lodder and van Putten, 2011).

The alpha peak was robustly identified in most regions and subjects. This is not the first attempt to assess the alpha peak spatial distribution of frequency and amplitude values in resting state, since clusters of alpha peaks in EEG recordings within a large sample of healthy population have been analyzed (Chiang et al., 2011). However, in the present study the MEG source space analysis allows a better understanding of the spatial distribution of this dominant alpha rhythm. Most studies of pathological aging have only focused on the posterior alpha peak (Osipova et al., 2006). Here we intentionally decided to consider sources of alpha rhythm other than the posterior ones, since alpha rhythms have been detected over wide regions of the brain [for a review, see Nunez et al. (2001)].

One of the main findings of our study is that the alpha rhythm of MCIs is slower when compared with a control population, especially over posterior regions. This is not surprising, since abnormally low alpha peak frequencies in AD have already been described (Passero et al., 1995). In the MCI literature less attention has been drawn to the alpha peak, but a reduced mean frequency score has been reported (Fernández et al., 2006). To gain further insight into the meaning of these peak alterations, their relationship with the hippocampal volume was considered. In fact, atrophy in medial temporal structures such as the hippocampus is a pathological marker of AD (Dubois et al., 2007; Prestia et al., 2013). Some studies have related a lower hippocampal volume to a higher delta and theta dipole density in AD (Fernández et al., 2003), lower power in the 8–10.5 Hz range (Babiloni et al., 2009), and an increase in the alpha3/alpha2 ratio (Moretti et al., 2009b). Our results show that hippocampal volumes correlated positively with peak frequencies in temporo-parieto-occipital regions of the brain and negatively with peak amplitude in occipital and frontal regions. This contributes to the idea that the peak frequency slowing is associated with a degenerative process, evolving in parallel with the loss of hippocampal volume. Two different hypotheses have been introduced over the past years to explain the increased low frequency power in AD and MCI. It could be explained through either (1) a slowing down or (2) a redistribution of the oscillatory sources in the theta-alpha frequency range (Osipova et al., 2005, 2006). This study supports the first hypothesis, although bigger samples and an analysis of the possible spatial shift of the sources would be needed to make stronger statements and investigate the second hypothesis.

The exact physiological origin of alpha rhythm remains unclear. Some studies indicate a prominent role of the thalamus (Hughes and Crunelli, 2005; Lőrincz et al., 2009; Bollimunta et al., 2011), while others point out the existence of cortical generators (Flint and Connors, 1996; Bollimunta et al., 2008). With a thalamo-cortical model of EEG generation, (Hindriks and van Putten, 2013) established that the resonance properties of cortico-thalamo-cortical, intra-cortical, and feedforward circuits determine alpha responses. They found that both a decreased firing of excitatory neuronal populations and an increased firing rate in inhibitory neuronal populations related to a decrease in alpha frequency. This modulation was particularly intense in the intra-cortical circuit: a decreased delay in this circuit produced a strong frequency slowing. Moreover, a decrease in the number of active synapses in thalamic nuclei could also explain an alpha power shift toward lower frequencies, as proved in a recent study with a thalamico-cortical-thalamic neural mass model (Bhattacharya et al., 2011). This model showed that the alpha frequency shift is especially sensitive to damage in inhibitory interneurons in the thalamus. Within this theory, the MCI alpha slowing found in this study would suggest that a synaptic damage is already present in the MCI stage. This in turn could be related with amyloid β, since its deposition has been shown to contribute to synaptic loss in AD (Reddy and Beal, 2008; Bate and Williams, 2011).

Additionally, the peak frequency is not determined exclusively by the pathology, but also depends on other factors like age or sex. In fact, we found a frequency decrease with age, and higher frequency values in females than in males. Such trends have been previously found in studies with large healthy samples (Chiang et al., 2011). In our study, we report that this trend is maintained in MCI patients. Most studies of sex differences in the alpha band have focused on childhood and young age, with mixed outcomes, some of them finding higher frequencies and earlier maturation in girls than boys (Petersén and Eeg-Olofsson, 2008). Our results also show higher frequencies in females than in males, although within a completely different age profile. Dustman et al. (1993) found that a slowing of alpha rhythms and an increase in delta, theta and beta activity are common age-associated changes in EEG spectra. This means that the alpha slowing is normal in healthy aging, and suggests that the MCI disease speeds up the natural aging process.

The methodological procedure followed here enabled the examination of amplitude and frequency shifts of the alpha peak. It combined beamforming of MEG resting state data, alpha peak fitting and ANOVA tests for statistical analysis, corrected for multiple comparisons with a procedure including clustering and permutations. Although it was tested with a rather small sample of subjects, it revealed a slowing of the alpha oscillatory sources in MCI and established that age, sex and hippocampal volume affect peak amplitude and frequency. However, larger samples would be needed to confirm these effects and to evaluate others, such as an interaction between age, sex, or educational level. Additionally, longitudinal follow-up studies could provide insight into the evolution of the slowing process and the onset of the AD-related pathology.

## Conclusion

In conclusion, we studied the spatial distribution of the alpha peak frequency and amplitude in a sample of controls and MCI patients using MEG resting state spectra in source space. Variations across subjects were found, even at a healthy stage, since peak frequency depended upon age and sex. The alpha peak was altered in the MCI sample when compared to controls: MCIs presented lower peak frequencies. This slowing of the alpha oscillatory sources in MCI could be attributed to an impaired thalamico-cortical circuit or a synaptic loss in thalamic populations. Furthermore, the peak frequency progression to lower frequencies correlated with the degree of hippocampal atrophy, highlighting its pathological meaning.

## Author contributions

Conception of the research: Fernando Maestú, Alberto Fernández, and Alberto Marcos. Neuropsychological assessment: Maria Emiliana de Andrés. MCI evaluation: Alberto Marcos. MRI data acquisition: Miguel Yus. Volumetric analysis: Jose Ángel Pineda-Pardo. MEG data acquisition and database organization: Maria Eugenia López and Sara Aurtenetxe. Methods design and data analysis: Pilar Garcés, Raul Vicente, Michael Wibral, and Miguel Sancho. Drafting of the manuscript: Pilar Garcés and Alberto Fernández. Critical revision of the manuscript: Alberto Fernández, Raul Vicente, Michael Wibral, and Fernando Maestú. Every author read and approved the final version of the manuscript.

### Conflict of interest statement

The authors declare that the research was conducted in the absence of any commercial or financial relationships that could be construed as a potential conflict of interest.

## References

[B1] Babiloni C. Carducci F. Lizio R. Vecchio F. Baglieri A. Bernardini S. (2013). Resting state cortical electroencephalographic rhythms are related to gray matter volume in subjects with mild cognitive impairment and Alzheimer's disease. Hum. Brain Mapp. 34, 1427–1446 10.1002/hbm.22005 22331654 PMC6869852

[B2] Babiloni C. Frisoni G. B. Pievani M. Vecchio F. Lizio R. Buttiglione M. (2009). Hippocampal volume and cortical sources of EEG alpha rhythms in mild cognitive impairment and Alzheimer disease. Neuroimage 44, 123–135 10.1016/j.neuroimage.2008.08.005 18805495

[B3] Bate C. Williams A. (2011). Amyloid-β -induced synapse damage is mediated via cross-linkage of cellular prion proteins. J. Biol. Chem. 286, 37955–37963 10.1074/jbc.M111.248724 21900234 PMC3207431

[B4] Berendse H. Verbunt J. P. Scheltens P. van Dijk B. Jonkman E. (2000). Magnetoencephalographic analysis of cortical activity in Alzheimer's disease: a pilot study. Clin. Neurophysiol. 111, 604–612 10.1016/S1388-2457(99)00309-0 10727911

[B5] Bhattacharya B. S. Coyle D. Maguire L. P. (2011). A thalamo-cortico-thalamic neural mass model to study alpha rhythms in Alzheimer's disease. Neural Netw. 24, 631–645 10.1016/j.neunet.2011.02.009 21435838

[B6] Bollimunta A. Chen Y. Schroeder C. E. Ding M. (2008). Neuronal mechanisms of cortical alpha oscillations in awake-behaving macaques. J. Neurosci. 28, 9976–9988 10.1523/JNEUROSCI.2699-08.2008 18829955 PMC2692971

[B7] Bollimunta A. Mo J. Schroeder C. E. Ding M. (2011). Neuronal mechanisms and attentional modulation of corticothalamic α oscillations. J. Neurosci. 31, 4935–4943 10.1523/JNEUROSCI.5580-10.2011 21451032 PMC3505610

[B8] Chiang A. K. I. Rennie C. J. Robinson P. A. Roberts J. A. Rigozzi M. K. Whitehouse R. W. (2008). Automated characterization of multiple alpha peaks in multi-site electroencephalograms. J. Neurosci. Methods 168, 396–411 10.1016/j.jneumeth.2007.11.001 18083237

[B9] Chiang A. K. I. Rennie C. J. Robinson P. A. van Albada S. J. Kerr C. C. (2011). Age trends and sex differences of alpha rhythms including split alpha peaks. Clin. Neurophysiol. 122, 1505–1517 10.1016/j.clinph.2011.01.040 21349761

[B10] Desikan R. S. Ségonne F. Fischl B. Quinn B. T. Dickerson B. C. Blacker D. (2006). An automated labeling system for subdividing the human cerebral cortex on MRI scans into gyral based regions of interest. Neuroimage 31, 968–980 10.1016/j.neuroimage.2006.01.021 16530430

[B11] Dubois B. Feldman H. H. Jacova C. Dekosky S. T. Barberger-Gateau P. Cummings J. (2007). Research criteria for the diagnosis of Alzheimer's disease: revising the NINCDS-ADRDA criteria. Lancet Neurol. 6, 734–746 10.1016/S1474-4422(07)70178-3 17616482

[B12] Dustman R. E. Shearer D. E. Emmerson R. Y. (1993). EEG and event-related potentials in normal aging. Prog. Neurobiol. 41, 369–401 10.1016/0301-0082(93)90005-D 8210412

[B13] Fernández A. Arrazola J. Maestú F. Amo C. Gil-Gregorio P. Wienbruch C. (2003). Correlations of hippocampal atrophy and focal low-frequency magnetic activity in Alzheimer disease: volumetric MR imaging-magnetoencephalographic study. AJNR Am. J. Neuroradiol. 24, 481–487 Available online at: http://www.ncbi.nlm.nih.gov/pubmed/12637301 12637301 PMC7973601

[B14] Fernández A. Hornero R. Mayo A. Poza J. Gil-Gregorio P. Ortiz T. (2006). MEG spectral profile in Alzheimer's disease and mild cognitive impairment. Clin. Neurophysiol. 117, 306–314 10.1016/j.clinph.2005.10.017 16386951

[B15] Fischl B. Salat D. H. Busa E. Albert M. Dieterich M. Haselgrove C. (2002). Whole brain segmentation. Neuron 33, 341–355 10.1016/S0896-6273(02)00569-X 11832223

[B16] Flint A. C. Connors B. W. (1996). Two types of network oscillations in neocortex mediated by distinct glutamate receptor subtypes and neuronal populations. J Neurophysiol 75, 951–957 8714667 10.1152/jn.1996.75.2.951

[B17] Grundman M. Petersen R. C. Ferris S. H. Thomas R. G. Aisen P. S. Bennett D. A. (2004). Mild cognitive impairment can be distinguished from Alzheimer disease and normal aging for clinical trials. Arch. Neurol. 61, 59–66 10.1001/archneur.61.1.59 14732621

[B18] Hindriks R. van Putten M. J. A. M. (2013). Thalamo-cortical mechanisms underlying changes in amplitude and frequency of human alpha oscillations. Neuroimage 70, 150–163 10.1016/j.neuroimage.2012.12.018 23266701

[B19] Huang C. Wahlund L.-O. Dierks T. Julin P. Winblad B. Jelic V. (2000). Discrimination of Alzheimer's disease and mild cognitive impairment by equivalent EEG sources: a cross-sectional and longitudinal study. Clin. Neurophysiol. 111, 1961–1967 10.1016/S1388-2457(00)00454-5 11068230

[B20] Hughes S. W. Crunelli V. (2005). Thalamic mechanisms of EEG alpha rhythms and their pathological implications. Neuroscientist 11, 357–372 10.1177/1073858405277450 16061522

[B21] Jasper H. Penfield W. (1949). Zur Deutung des normalen Elektrencephalogramms und selner Veriinderungen. Electroeorticograms in man: effect of voluntary movement upon the electrical activity of the preeentral gyrus *. Arch. Psychiatr. Z. Neurol. 174, 163–174 10.1007/BF01062488

[B22] Jenkinson M. Beckmann C. F. Behrens T. E. J. Woolrich M. W. Smith S. M. (2012). FSL. Neuroimage 62, 782–790 10.1016/j.neuroimage.2011.09.015 21979382

[B23] Lodder S. S. van Putten M. J. A M. (2011). Automated EEG analysis: characterizing the posterior dominant rhythm. J. Neurosci. Methods 200, 86–93 10.1016/j.jneumeth.2011.06.008 21722667

[B24] Lőrincz M. L. Kékesi K. A. Juhász G. Crunelli V. Hughes S. W. (2009). Temporal framing of thalamic relay-mode firing by phasic inhibition during the alpha rhythm. Neuron 63, 683–696 10.1016/j.neuron.2009.08.012 19755110 PMC2791173

[B25] Maris E. Oostenveld R. (2007). Nonparametric statistical testing of EEG- and MEG-data. J. Neurosci. Methods 164, 177–190 10.1016/j.jneumeth.2007.03.024 17517438

[B26] Moretti D. V. Fracassi C. Pievani M. Geroldi C. Binetti G. Zanetti O. (2009a). Increase of theta/gamma ratio is associated with memory impairment. Clin. Neurophysiol. 120, 295–303 10.1016/j.clinph.2008.11.012 19121602

[B27] Moretti D. V. Pievani M. Fracassi C. Binetti G. Rosini S. Geroldi C. (2009b). Increase of theta/gamma and alpha3/alpha2 ratio is associated with amygdalo-hippocampal complex atrophy. J. Alzheimers Dis. 17, 349–357 10.3233/JAD-2009-1059 19363263

[B28] Moretti D. V. Frisoni G. B. Fracassi C. Pievani M. Geroldi C. Binetti G. (2011). MCI patients' EEGs show group differences between those who progress and those who do not progress to AD. Neurobiol. Aging 32, 563–571 10.1016/j.neurobiolaging.2009.04.003 20022139

[B29] Moretti D. V. Paternicò D. Binetti G. Zanetti O. Frisoni G. B. (2012). EEG markers are associated to gray matter changes in thalamus and basal ganglia in subjects with mild cognitive impairment. Neuroimage 60, 489–496 10.1016/j.neuroimage.2011.11.086 22166796

[B30] Nolte G. (2003). The magnetic lead field theorem in the quasi-static approximation and its use for magnetoencephalography forward calculation in realistic volume conductors. Phys. Med. Biol. 48, 3637–3652 10.1088/0031-9155/48/22/002 14680264

[B31] Nunez P. L. Wingeier B. M. Silberstein R. B. (2001). Spatial-temporal structures of human alpha rhythms: theory, microcurrent sources, multiscale measurements, and global binding of local networks. Hum. Brain Mapp. 13, 125–164 10.1002/hbm.1030 11376500 PMC6872048

[B32] Oostenveld R. Fries P. Maris E. Schoffelen J.-M. (2011). FieldTrip: open source software for advanced analysis of MEG, EEG, and invasive electrophysiological data. Comput. Intell. Neurosci. 2011, 156869 10.1155/2011/156869 21253357 PMC3021840

[B33] Osipova D. Ahveninen J. Jensen O. Ylikoski A. Pekkonen E. (2005). Altered generation of spontaneous oscillations in Alzheimer's disease. Neuroimage 27, 835–841 10.1016/j.neuroimage.2005.05.011 15961323

[B34] Osipova D. Rantanen K. Ahveninen J. Ylikoski R. Häppölä O. Strandberg T. (2006). Source estimation of spontaneous MEG oscillations in mild cognitive impairment. Neurosci. Lett. 405, 57–61 10.1016/j.neulet.2006.06.045 16854528

[B35] Passero S. Rocchi R. Vatti G. Burgalassi L. Battistini N. (1995). Quantitative EEG mapping, regional cerebral blood flow, and neuropsychological function in Alzheimer's disease. Dement. Geriatr. Cogn. Disord. 6, 148–156 10.1159/000106938 7620527

[B36] Petersén I. Eeg-Olofsson O. (2008). The development of the electroencephalogram in normal children from the age of 1 through 15 years – non-paroxysmal activity. Neuropediatrics 2, 247–304 10.1055/s-0028-1091786 5171359

[B37] Petersen R. C. (2001). Current concepts in mild cognitive impairment. Arch. Neurol. 58, 1985–1992 10.1001/archneur.58.12.1985 11735772

[B38] Petersen R. C. Negash S. (2008). Mild cognitive impairment: an overview. CNS Spectr. 13, 45–53 Available online at: http://www.ncbi.nlm.nih.gov/pubmed/18204414 18204414 10.1017/s1092852900016151

[B39] Prestia A. Caroli A. van der Flier W. M. Ossenkoppele R. Van Berckel B. Barkhof F. (2013). Prediction of dementia in MCI patients based on core diagnostic markers for Alzheimer disease. Neurology 80, 1048–1056 10.1212/WNL.0b013e3182872830 23390179

[B40] Reddy P. H. Beal M. F. (2008). Amyloid beta, mitochondrial dysfunction and synaptic damage: implications for cognitive decline in aging and Alzheimer's disease. Trends Mol. Med. 14, 45–53 10.1016/j.molmed.2007.12.002 18218341 PMC3107703

[B41] Rosen W. G. Terry R. D. Fuld P. A. Katzman R. Peck A. (1980). Pathological verification of ischemic score in differentiation of dementias. Ann. Neurol. 7, 486–488 10.1002/ana.410070516 7396427

[B42] Samson-Dollfus D. Delapierre G. Do Marcolino C. Blondeau C. (1997). Normal and pathological changes in alpha rhythms. Int. J. Psychophysiol. 26, 395–409 10.1016/S0167-8760(97)00778-2 9203017

[B43] Taulu S. Simola J. (2006). Spatiotemporal signal space separation method for rejecting nearby interference in MEG measurements. Phys. Med. Biol. 51, 1759–1768 10.1088/0031-9155/51/7/008 16552102

[B44] Van Veen B. D. van Drongelen W. Yuchtman M. Suzuki A. (1997). Localization of brain electrical activity via linearly constrained minimum variance spatial filtering. IEEE Trans. Biomed. Eng. 44, 867–880 10.1109/10.623056 9282479

[B45] Yesavage J. A. Brink T. L. Rose T. L. Lum O. Huang V. Adey M. (1982). Development and validation of a geriatric depression screening scale: a preliminary report. J. Psychiatr. Res. 17, 37–49 10.1016/0022-3956(82)90033-4 7183759

